# Management of Postpartum Preeclampsia and Hypertensive Disorders (MOPP): Postpartum Tight vs Standard Blood Pressure Control

**DOI:** 10.1016/j.jacadv.2025.101617

**Published:** 2025-01-31

**Authors:** Emily B. Rosenfeld, Deepika Sagaram, Rachel Lee, Ernani Sadural, Richard C. Miller, Ruby Lin, Deshae Jenkins, Kristin Blackledge, Ivana Nikodijevic, Alex Rizzo, Vanessa Martinez, Emily E. Daggett, Olivia McGeough, Cande V. Ananth, Todd Rosen

**Affiliations:** a Division of Maternal-Fetal Medicine, Department of Obstetrics, Gynecology, and Reproductive Sciences, Rutgers Robert Wood Johnson Medical School, New Brunswick, New Jersey, USA; b Division of Epidemiology and Biostatistics, Department of Obstetrics, Gynecology, and Reproductive Sciences, Rutgers Robert Wood Johnson Medical School, New Brunswick, New Jersey, USA; c Department of Obstetrics and Gynecology, Cooperman Barnabas Medical Center, RWJBarnabas Health, Livingston, New Jersey, USA; d Rutgers Robert Wood Johnson Medical School, New Brunswick, New Jersey, USA; e New Jersey Medical School, Newark, New Jersey, USA; f Cardiovascular Institute of New Jersey, Rutgers Robert Wood Johnson Medical School, New Brunswick, New Jersey, USA; g Department of Medicine, Rutgers Robert Wood Johnson Medical School, New Brunswick, New Jersey, USA; h Department of Biostatistics and Epidemiology, Rutgers School of Public Health, Piscataway, New Jersey, USA; i Environmental and Occupational Health Sciences Institute, Rutgers Robert Wood Johnson Medical School, Piscataway, New Jersey, USA

**Keywords:** hypertension, postpartum, preeclampsia

## Abstract

**Background:**

It is unknown whether tightly controlled blood pressure in the postpartum period will improve outcomes.

**Objectives:**

The purpose of this study was to assess the effect of a lower treatment threshold (≥130/80 mm Hg) for initiating and titrating antihypertensive medication on reducing emergency department visits in postpartum patients with hypertension.

**Methods:**

A prospective cohort of postpartum patients was recruited in a multicenter study between March 2023 and March 2024 and treated to maintain blood pressure <130/80 mm Hg using remote blood pressure monitoring. These patients were compared to a propensity score-matched retrospective cohort from February 2021 to February 2023 who were treated to maintain blood pressures <150/100 mm Hg. Eligible patients were 18 or older with a diagnosis of hypertensive disorder. The primary outcome was an emergency department visit for hypertension.

**Results:**

There were 392 patients enrolled in the interventional cohort and 1,204 patients identified in the retrospective cohort. After the propensity score match, 276 and 429 patients remained in the prospective and retrospective groups, respectively. Emergency department visits for hypertensive disorders occurred in 10 patients (3.6%) in the intervention and 36 patients (8.4%) in the retrospective cohort (risk difference −4.8; 95% CI: −8.2 to −1.3; doubly robust OR: 0.32; 95% CI: 0.10-1.01). At 6 weeks postpartum, compared to the retrospective group, the intervention group had systolic and diastolic blood pressure that was 4.4 mm Hg (95% CI: −6.8 to −2.0) and 3.1 mm Hg (95% CI: −4.9 to −1.2) lower, respectively.

**Conclusions:**

Tighter blood pressure control was associated with reduced postpartum emergency department visits for hypertensive disorders.

In the United States, 3% to 6% of all pregnancies are complicated by preeclampsia, and 5% to 12% of those patients return to the hospital with hypertensive disorders when they are postpartum.^1^^,^^2^ Black pregnant persons are especially vulnerable to antepartum and postpartum preeclampsia and have higher rates of morbidity and mortality compared to their White counterparts.^3^ The prevalence of hypertension is increasing,^4^ and it is one of the most substantial modifiable risk factors for cardiovascular disease.^5^ Tight blood pressure control in the postpartum period may decrease the risk of cardiovascular disease later in life.^6^^,^^7^

The current guidelines from the American College of Obstetricians and Gynecologists for managing postpartum hypertension are based on a 1987 case series of 67 patients and recommend a cutoff of 150/100 mm Hg to start antihypertensive medications in the postpartum period.^8^, ^9^, ^10^ Outside of pregnancy, new guidelines and recommendations for blood pressure control have shown that a reduction in blood pressure decreases cardiovascular events.^11^ As a result of these findings, the 2017 American College of Cardiology and American Heart Association (AHA) Blood Pressure Guidelines recommend systolic and diastolic blood pressure treatment goals of less than 130 mm Hg and less than 80 mm Hg, respectively, for nonpregnant adults on antihypertensive medication.^12^

Tita et al^13^ reported that tight blood pressure control to <140/90 mm Hg during the antepartum period for patients with chronic hypertension improved maternal and neonatal outcomes. Limited studies have evaluated the effects of a lower blood pressure target and stricter blood pressure control during the postpartum period to reduce complications of preeclampsia and its severe sequelae. However, without the safety concerns for the fetus, the postpartum period presents an optimal opportunity to assess if tighter blood pressure control may be beneficial.

More than 60% of maternal deaths occur during the postpartum period, of which hypertension is a major contributor to these deaths.^14^ Postpartum blood pressure most typically reaches a maximum at 4 to 6 days postpartum^15^ and returns to the prepregnancy state around 4 to 6 weeks postpartum.^16^ Patient self-monitoring of blood pressure has revealed that more than 80% of patients with hypertensive disorders of pregnancy have persistently elevated blood pressure.^17^ Remote patient monitoring offers an innovative approach to blood pressure assessment and titration of medication for patients who do not have the resources to make frequent in-person visits.^18^, ^19^, ^20^ However, in one study, the increased surveillance led to increased emergency department visits,^21^ which may have been because no adjustments in therapy were accompanied by a change in monitoring. Additional emergency department visits or postpartum admissions can impact mother-infant bonding, breastfeeding, and mental health, in addition to driving up the cost of health care.^22^^,^^23^

Hypertensive disorders in pregnancy, particularly preeclampsia, are strong risk factors for cardiovascular disease along the life course.^24^^,^^25^ It is plausible that reducing blood pressure through titrated medication and dosage would mitigate these cardiovascular disease risks. We hypothesized that persons with high blood pressure with an activated medication titration regimen would have reduced emergency department visits. We tested this hypothesis in a large cohort of postpartum persons using a tight blood pressure control algorithm (goal blood pressure <130/80 mm Hg) and remote blood pressure to reduce postpartum emergency department visits for hypertensive disorders.

## Methods

### Trial design

A prospective cohort of patients who utilized remote patient monitoring to achieve tight blood pressure control was compared to a retrospective cohort of patients with standard blood pressure management using a propensity score methodology. This trial was conducted at 2 tertiary hospitals, Rutgers Robert Wood Johnson University Hospital, New Brunswick, NJ, and Cooperman Barnabas Medical Center, Livingston, NJ. Rutgers University maintained the Institutional Board Approval of record, and the study was registered with ClinicalTrials.gov (NCT05775744). The prospective cohort included patients who delivered between March 28, 2023, and March 26, 2024, and they were compared to a retrospective cohort with deliveries between February 1, 2021, and February 1, 2023. Study data were collected and managed using REDCap electronic data capture tools hosted at Rutgers University.^26^^,^^27^ The study followed the TREND (Transparent Reporting of Evaluations with Nonrandomized Design) reporting guidelines for nonrandomized studies.^28^

### Eligibility

**Figure 1:**
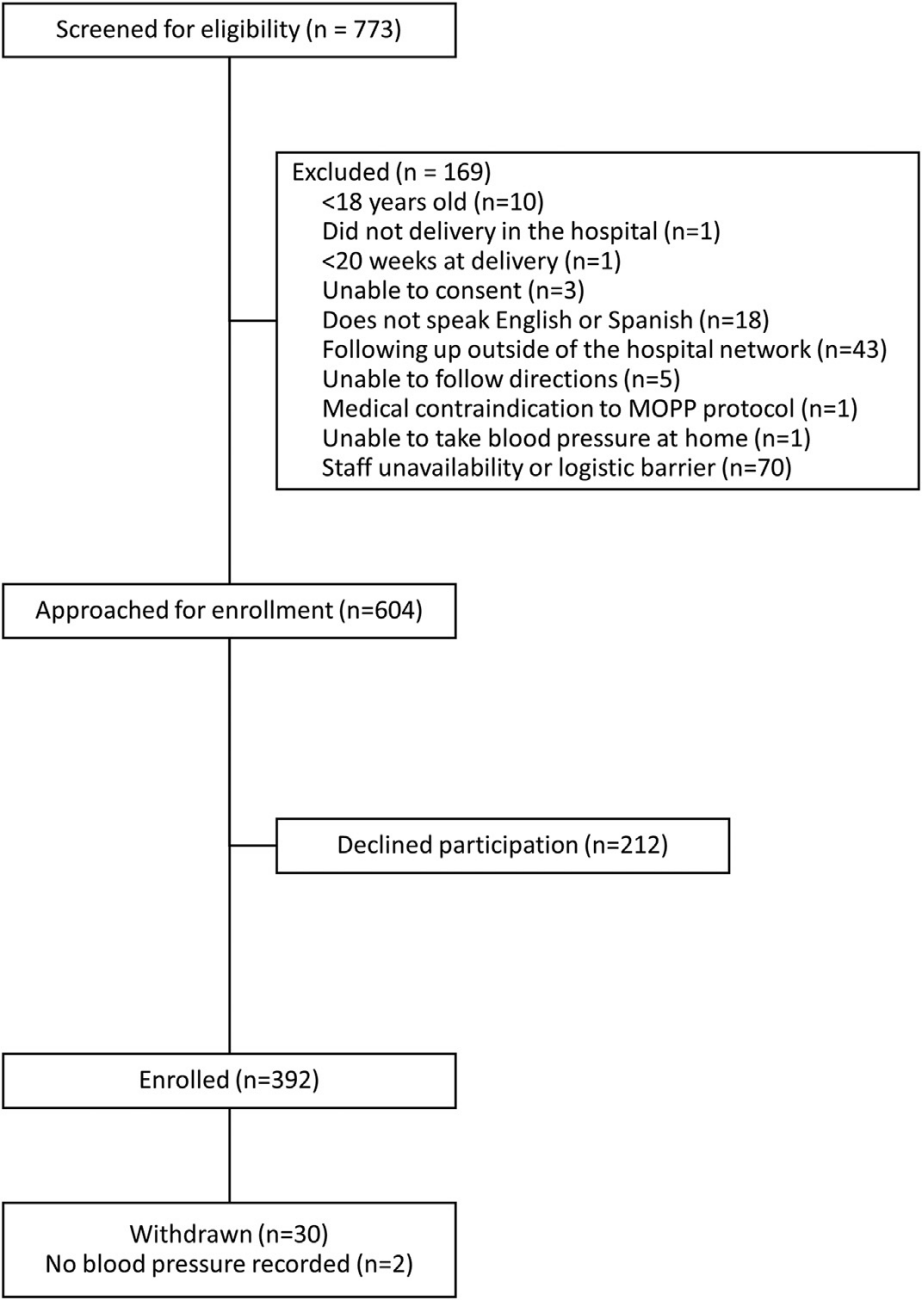
Transparent Reporting of Evaluations With Nonrandomized Design (TREND) Diagram MOPP = Management of Postpartum Preeclampsia and Hypertensive Disorders.

Patients were included if they were 18 or older and delivered at 20 weeks gestation or beyond during the current hospitalization. All subjects met the criteria for hypertensive disorders of pregnancy (chronic hypertension, gestational hypertension, or preeclampsia).^8^^,^^29^ The prospective cohort was also required to consent, speak English or Spanish, follow-up with a physician associated with the hospital system, and be able to follow study protocols. Potential study subjects in the prospective arm were excluded if they had any medical condition that the providers felt was a contraindication to the treatment algorithm or were unwilling to monitor their blood pressure at home. Of the 657 patients screened, 392 were enrolled in the prospective arm (Figure 1). Billing codes were used to identify the patients in the retrospective arm of the study (International Classification of Diseases, version 10 codes O10-O16).

### Intervention and procedures

Patients were approached and enrolled postpartum before discharge from their delivery hospitalization. Patients enrolled in the prospective arm were prescribed blood pressure medication during hospitalization to maintain a blood pressure <130/80 mm Hg. Before discharge, the patients were set up with a remote patient monitoring system and were instructed on the correct technique for measuring blood pressure. Discharge timing was left to the discretion of the admitting physician. Blood pressure readings were transferred by Bluetooth to the Health Recovery Solutions platform and were monitored by a physician at all hours of the day.

In response to recent publications, nifedipine extended release was preferentially used over labetalol,^30^^,^^31^ but patients could be continued on labetalol or restarted on their prepregnancy antihypertensive medication. If patients had blood pressure ≥130/80 mm Hg, nifedipine extended release was started at 30 mg or increased by 30 mg to reach a target blood pressure of <130/80 mm Hg. Labetalol was titrated by 200 mg twice a day or 100 mg 3 times a day. Medication was decreased by one titration unit for blood pressure that was persistently <110/80 mm Hg and discontinued for blood pressure persistently <100/80 mm Hg (Supplemental Table 1). A single medication was increased to the highest tolerated dose before starting a second medication to maximize compliance.

Enrolled subjects measured their blood pressure twice daily for the first 72 hours after discharge and daily after that. Subjects were instructed to repeat their blood pressure after any abnormal readings. Patients who were normotensive for 1 week, not on medication, and greater than 2 weeks from discharge decreased the frequency of blood pressure readings to once a week. If it had been more than 72 hours from an expected blood pressure reading, study staff would call to remind the patient to take their blood pressure. After 6 weeks, remote monitoring equipment was returned, and blood pressure management was transitioned to the patient’s primary provider. The retrospective cohort did not have a uniform protocol for starting or titrating antihypertensive medications, but the clinical standard during this time was to titrate antihypertensive medication to a goal of <150/100 mm Hg.

### Outcomes

The primary outcome was emergency department visits for hypertensive disorders. Secondary outcomes included postpartum readmissions for hypertensive disorders, blood pressure in the postpartum period, and attendance at postpartum visits.

### Statistical analysis

The estimated rate of emergency department visits for patients with hypertensive disorders was 12%. It was determined that 392 patients were needed to enroll in the prospective arm to provide 80% power to detect a 50% decrease in the primary outcome (assuming a 10% loss to follow-up and a 2-tailed type I error rate of 0.05).

Multiple imputations were undertaken for missing covariate data, with the pattern of missing data assumed to be “missing at random.” Fifty imputed data sets were created based on the fully conditional specification method,^32^ with the imputation models comprising all covariates. The standard errors were pooled and combined using Rubin’s principle.^33^

A propensity score analysis was performed using the greedy matching algorithm^34^ to develop pseudorandomized groups of tight blood pressure control vs usual care. Logistic regression models were used to estimate the propensity scores using covariates as predictors. Hosmer-Lemeshow goodness-of-fit was used, and no model violations were apparent. A matching algorithm created a 3-to-1 match (without replacement) of usual care vs tight blood pressure control (caliper distance of <0.1). Main effects at baseline included race/ethnicity, parity, marital status, body mass index at delivery, payer, type of hypertensive disorder, aspirin use, diabetes, type of labor, type of delivery, multifetal gestation, maternal lupus, language spoken, maternal age at delivery, gestational age at delivery, and use of magnesium sulfate for preeclampsia. Two-way interactions between maternal race with body mass index, type of hypertensive disorder, diabetes, and gestational age and between the type of hypertensive disorder with aspirin, diabetes, gestational age, type of labor, and magnesium sulfate for preeclampsia were included. We also considered other potential 2-way interactions not included in the final model. Since these interaction terms were not predictive of tight blood pressure control, these terms were dropped from the final model. The approach was to develop the best parsimonious prediction model based on available covariates. To account for institutional differences, patients from the prospective group were matched to retrospective controls from the same institution.

An intent-to-treat approach was used to estimate the risk differences (RD) and the matched pair OR with their corresponding 95% CI in the primary and secondary outcomes. In addition, doubly robust OR was reported after adjusting for confounding covariates listed in the prediction model. As a sensitivity analysis, we also performed a per-protocol analysis of the primary outcome.

### Sensitivity analysis

Since the matching algorithm could not identify matches for 116 patients in the prospective arm, the primary analysis was replicated based on the inverse probability of the treatment weighting method. The results of the inverse probability of treatment weighting weighted propensity score match are shown in Supplemental Table 2.

Statistical analysis was performed in SAS (version 9.4, SAS Institute) and visualization using R (version 4.3.2, R Foundation for Statistical Computing).

## Results

From the source population of 392 patients in the prospective intervention arm and 1,204 patients in the retrospective control arm, 276 patients in the intervention arm were matched to 429 patients in the control group. Characteristics of the original cohort and propensity score matched cohort are described in Table 1.

**Table 1:** Distribution of Maternal and Neonatal Characteristics of the Original and Propensity Score-Matched Cohorts of Tight vs Standard Blood Pressure Control

	Original Cohort (n = 1,596)		Propensity Scores Matched Groups (n = 705)	
	Tight Blood Pressure Control (n = 392)	Standard Blood Pressure Control (n = 1,204)	Tight Blood Pressure Control (n = 276)	Standard Blood Pressure Control (n = 429)
Maternal age at delivery	32.2 ± 6.0	33.0 ± 5.8	32.0 ± 6.1	32.5 ± 6.1
Gestational age at delivery				
Median	37.6 ± 2.7	36.9 ± 3.1	37.3 ± 2.8	37.0 ± 3.2
<28 wk	6 (1.5)	34 (2.8)	4 (1.5)	14 (3.3)
<34 wk	30 (7.7)	164 (13.6)	27 (9.8)	56 (13.1)
<37 wk	303 (77.3)	746 (62.0)	198 (71.7)	278 (64.8)
≥37 wk	89 (22.7)	458 (38.0)	78 (28.3)	151 (35.2)
Race or ethnicity				
Non-Hispanic White	114 (29.1)	251 (20.9)	79 (28.6)	112 (26.1)
Non-Hispanic Black	84 (21.4)	162 (13.5)	54 (19.6)	90 (21.0)
Hispanic	137 (35.0)	238 (19.8)	98 (35.5)	148 (34.5)
Asian/Pacific Islander	25 (6.4)	80 (6.6)	20 (7.3)	38 (8.9)
Other	31 (7.9)	111 (9.2)	25 (9.1)	41 (9.6)
Unknown	1 (0.3)	362 (30.1)	—	—
Parity				
Nulliparous	135 (40.5)	468 (45.3)	117 (42.4)	192 (44.8)
One prior delivery	97 (29.1)	306 (29.6)	79 (28.6)	115 (26.8)
≥2 deliveries	101 (30.3)	259 (25.1)	80 (29.0)	122 (28.4)
Marital status				
Single	171 (43.6)	420 (35.1)	130 (47.1)	205 (47.8)
Married	221 (56.4)	776 (64.9)	146 (52.9)	224 (52.2)
Language spoken				
English	340 (86.7)	1,077 (90.0)	233 (84.4)	359 (83.7)
Spanish	52 (13.3)	120 (10.0)	43 (15.6)	70 (16.3)
Body mass index (kg/m^2^) at delivery				
Mean (SD)	34.9 ± 7.0	34.5 ± 7.2	34.6 ± 7.3	35.1 ± 7.3
<30	93 (23.9)	279 (27.8)	73 (26.5)	99 (23.1)
30-39	216 (55.5)	531 (52.9)	151 (54.7)	235 (54.8)
≥40	80 (20.6)	194 (19.3)	52 (18.8)	95 (22.1)
Insurance status				
Private payer	169 (43.2)	299 (24.9)	146 (52.9)	237 (55.2)
Government sponsored	216 (55.2)	860 (71.6)	125 (45.3)	187 (43.6)
Self-pay	5 (1.3)	27 (2.3)	5 (1.8)	5 (1.2)
Other	1 (0.3)	16 (1.3)	—	—
Missing data	1 (0.3)	2 (0.2)	—	—
Risk factors during pregnancy				
Smoking	11 (2.8)	18 (1.5)	10 (3.6)	5 (1.2)
Alcohol use	2 (0.5)	5 (0.4)	2 (0.7)	3 (0.7)
Substance use	13 (3.3)	15 (1.6)	11 (4.0)	8 (1.9)
Aspirin use	158 (40.3)	588 (49.2)	113 (40.9)	162 (37.8)
Maternal lupus	5 (1.3)	5 (0.4)	4 (1.5)	3 (0.7)
Multifetal gestations	14 (3.6)	63 (5.2)	10 (3.6)	18 (4.2)
Diabetes	86 (22.1)	242 (20.1)	62 (22.5)	87 (20.3)
Prior to delivery admission				
Taking antihypertensive medication	69 (17.7)	183 (15.2)	52 (18.8)	78 (18.2)
Mean gestational age at highest BP	30.2 ± 9.2	29.3 ± 9 0.3	30.2 ± 9.2	31.3 ± 8.6
Mean highest systolic BP	140.4 ± 14.9	143.6 ± 18.3	141.8 ± 15.6	147.8 ± 19.0
Mean highest diastolic BP	84.5 ± 10.0	87.1 ± 11.4	85.2 ± 10.4	88.3 ± 11.5
Type of labor				
None	106 (27.3)	380 (31.6)	62 (22.5)	111 (25.9)
Induced	209 (53.7)	626 (52.0)	157 (56.9)	223 (52.0)
Spontaneous	74 (19.0)	198 (16.5)	57 (20.7)	95 (22.1)
Type of delivery				
Spontaneous vaginal	189 (48.5)	538 (44.7)	139 (50.4)	201 (46.9)
Operative vaginal	24 (6.2)	45 (3.7)	15 (5.4)	19 (4.4)
Cesarean	177 (45.4)	621 (51.6)	122 (44.2)	209 (48.7)
Hypertensive disorder				
Chronic hypertension	56 (14.3)	40 (3.3)	36 (13.0)	38 (8.9)
Chronic hypertension with superimposed preeclampsia	44 (11.2)	256 (21.3)	38 (13.8)	92 (21.5)
Preeclampsia	136 (34.7)	785 (65.2)	125 (45.3)	221 (51.5)
Gestational hypertension	156 (39.8)	123 (10.2)	77 (27.9)	78 (18.2)
Feature of preeclampsia				
Proteinuria	101 (25.8)	223 (18.5)	89 (32.3)	74 (17.3)
Severe range BP	149 (38.0)	939 (78.0)	133 (48.2)	275 (64.1)
Headache	20 (5.1)	62 (5.2)	18 (6.5)	18 (4.2)
Visual symptoms	6 (1.5)	31 (2.6)	6 (2.2)	8 (1.9)
Thrombocytopenia	8 (2.0)	44 (3.7)	8 (2.9)	9 (2.1)
Transaminitis	15 (3.8)	88 (7.3)	13 (4.7)	20 (4.7)
Pulmonary edema	2 (0.5)	2 (0.2)	2 (0.7)	1 (0.2)
Eclampsia	2 (0.5)	1 (0.1)	2 (0.7)	0
Renal injury	5 (1.3)	43 (3.6)	5 (1.8)	7 (1.6)
Magnesium sulfate	156 (84.8)	1,040 (95.7)	142 (51.5)	284 (66.2)
ICU admission	2 (0.5)	11 (0.9)	1 (0.4)	4 (0.9)
Neonatal outcome^a^				
Live birth	375 (99.2)	1,124 (98.5)	264 (99.3)	403 (98.1)
Stillbirth	2 (0.5)	11 (1.0)	2 (0.8)	5 (1.2)
Neonatal death	0	6 (0.5)	0	3 (0.7)
Elective termination	1 (0.3)	0	0	0
Male fetal sex^a^	193 (51.1)	578 (50.8)	133 (50.0)	208 (50.7)
APGAR score^a^				
1 min	9 (8-9)	8 (7-9)	8 (8-9)	9 (7-9)
5 min	9 (9-9)	9 (9-9)	9 (9-9)	9 (9-9)
Highest level of neonatal care^a^				
Well baby nursery	260 (68.8)	721 (63.4)	178 (66.9)	256 (62.6)
NICU	115 (30.4)	403 (35.4)	86 (32.3)	148 (36.2)
Not applicable	3 (0.8)	14 (1.2)	2 (0.8)	5 (1.2)
Neonatal birthweight	2,966 ± 723	2,780 ± 786	2,898 ± 740	2,782 ± 797

Values are mean ± SD, n (%), or median (IQR).

BP = blood pressure; ICU = intensive care unit; NICU = neonatal intensive care unit.

a Multifetal gestations are not included.

Before delivery admission, in the intervention cohort, 18.8% of patients were taking antihypertensive medication. The mean highest blood pressure during pregnancy was 141.8 ± 15.6/85.2 ± 10.4 mm Hg and occurred at 30.2 ± 9.2 weeks gestation. In the control cohort, 18.2% of patients were on antihypertensive medication before delivery admission. The mean highest blood pressure was 147.8 ± 19.0/88.3 ± 11.5 mm Hg occurring at 31.3 ± 8.6 weeks gestation. Chronic hypertension was recorded in 13.0% and 8.9%, chronic hypertension with superimposed preeclampsia in 13.8% and 21.5%, preeclampsia in 45.3% and 51.5%, and gestational hypertension in 27.9% and 18.2% of the intervention group and control group, respectively. Neonatal outcomes were similar between the 2 groups (Table 1).

**Figure 2:**
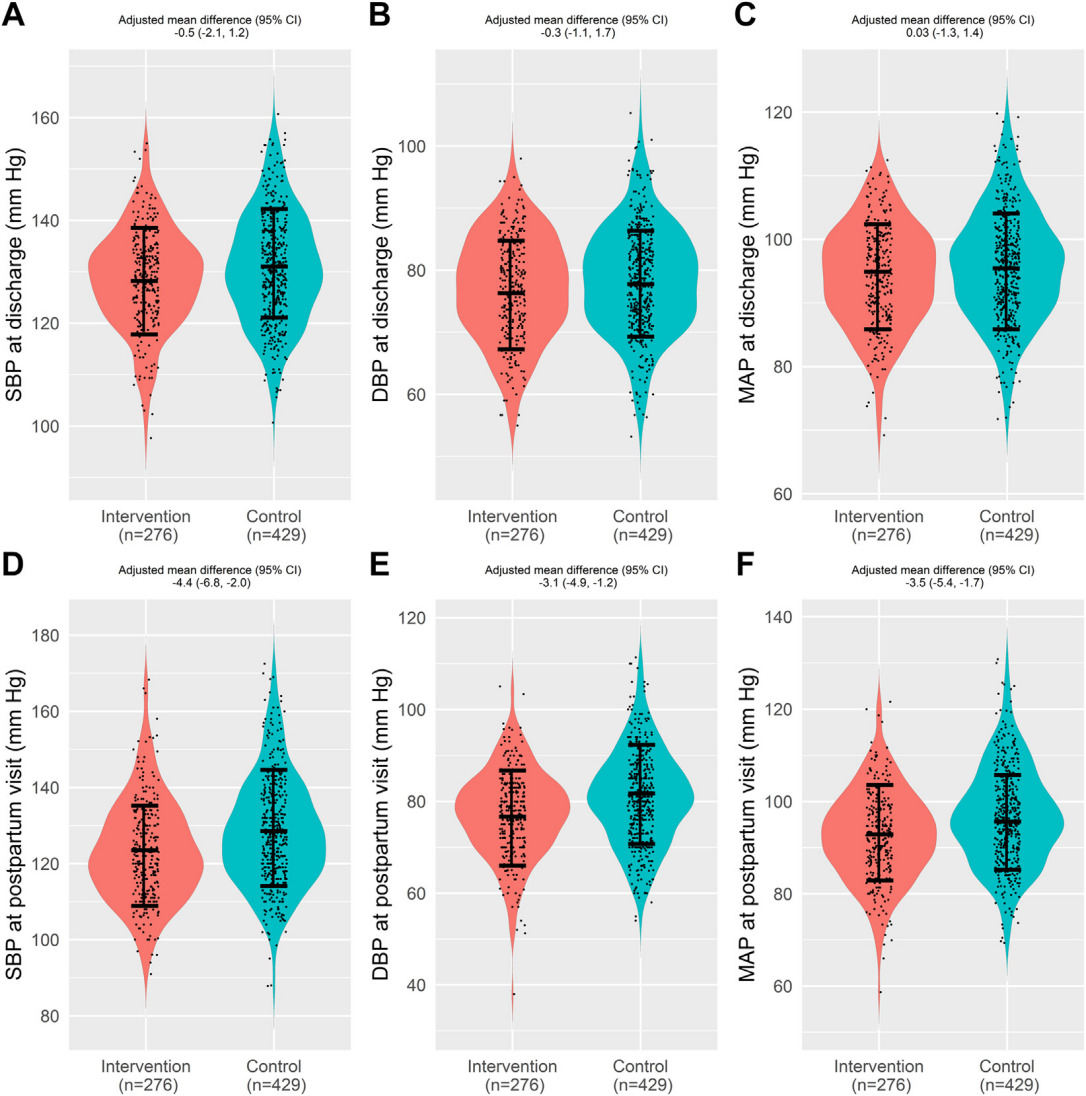
Violin Plot of Systolic and Diastolic Blood Pressures and Mean Arterial Pressure Readings, With Mean ± SD, and Adjusted Mean Difference With 95% CIs at Discharge and 6 Weeks Postpartum Comparing the Intervention and Control Groups DBP = diastolic blood pressure; MAP = mean arterial blood pressure; SBP = systolic blood pressure.

Emergency department visits with or without hospitalization for hypertensive disorders occurred in 10 patients (3.6%) in the intervention cohort and 36 patients (8.4%) in the control group (RD: −4.8; 95% CI: −8.2, −1.3; doubly robust OR: 0.32; 95% CI: 0.10, 1.01; Table 2 and Central Illustration). When the primary outcome of emergency department visits was evaluated as a per-protocol analysis, the results were similar (RD: −4.8, 95% CI: −8.3 to −1.3; doubly robust OR: 0.29; 95% CI: 0.08-1.05). In the intent-to-treat analysis, the intervention group had a lower adjusted mean difference in systolic blood pressure (−4.4, 95% CI: −6.8 to −2.0), diastolic blood pressure (−3.1, 95% CI: −4.9 to −1.2), and mean arterial pressure (−3.5, 95% CI: −5.4 to −1.7) at 6 weeks postpartum (Figure 2, Supplemental Table 3). There was a lower mean systolic and diastolic blood pressure and mean arterial pressure throughout the 6 weeks postpartum in the intervention group compared to the control group (Central Illustration). Blood pressure remained lower in the intervention group regardless of the type of hypertensive disorder of pregnancy (Supplemental Figure 1).

**Table 2:** Risks of Adverse Maternal Outcomes Among the Tight Blood Pressure Control and Standard Blood Pressure Control Groups Based on the Propensity Score-Matched Analysis

Maternal Outcomes	Tight Blood Pressure Control (n = 276)	Standard Blood Pressure Control (n = 429)	Risk Difference (95% CI)	OR (95% CI)	Doubly Robust OR (95% CI)
Emergency room visit for hypertensive disorders (with or without admission)	10 (3.6)	36 (8.4)	−4.8 (−8.2 to −1.3)	0.39 (0.17-0.91)	0.32 (0.10-1.01)
Discharged on postpartum day	2.7 ± 1.3	2.8 ± 1.4	—	—	—
Antihypertensive medication at discharge	202 (73.19)	199 (46.4)	26.8 (19.8-33.8)	3.71 (2.50-5.53)	6.89 (3.81-12.4)
Labetalol	65 (23.6)	126 (29.4)	−5.8 (−12.4 to 0.8)	0.78 (0.54-1.14)	0.78 (0.48-1.29)
Nifedipine extended release	168 (60.9)	118 (27.5)	33.3 (26.2-40.5)	5.11 (3.38-7.73)	8.78 (4.93-15.6)
Other	4 (1.5)	6 (1.4)	1.1 (−1.6 to 3.7)	1.57 (0.64-3.87)	—
Postpartum visit attendance					
Attended postpartum visits	196 (71.0)	233 (54.3)	—	1.47 (0.70-3.10)	1.37 (0.36-5.24)
Did not attend postpartum visit	18 (6.5)	36 (8.4)	—	—	—
Unknown	62 (22.5)	160 (37.3)	—	—	—
Currently prescribed antihypertensive medication at postpartum visit	109 (55.9)	122 (54.0)	1.9 (−7.6 to 11.4)	1.27 (0.76-2.11)	1.48 (0.63-3.50)
Hospital readmission for hypertensive disorders	4 (1.5)	20 (4.7)	−3.2 (−5.7 to −0.8)	0.24 (0.06-0.99)	0.09 (0.00-2.31)
Unanticipated hospital visit					
Antihypertensive medication adjustment	9 (3.3)	30 (7.0)	−3.7 (−6.9 to −0.5)	—	—
Magnesium sulfate	3 (1.1)	15 (3.5)	−2.4 (−4.5 to −0.3)	—	—
Emergency department visit for any reason (with or without hospitalization)	36 (13.0)	67 (15.6)	−2.6 (−7.8 to 2.7)	0.79 (0.49-1.29)	0.78 (0.45-1.35)
Hospital readmission for any reason	13 (4.7)	28 (6.5)	−1.8 (−5.2 to 1.6)	0.60 (0.27-1.32)	0.36 (0.08-1.59)
Patients withdrawn	25 (9.1)	—	—	—	—

**Central Illustration:**
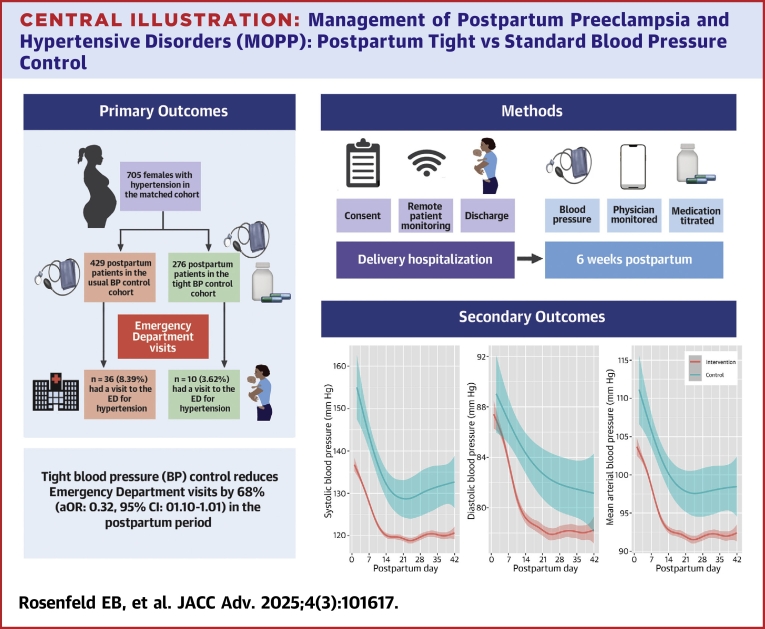
Management of Postpartum Preeclampsia and Hypertensive Disorders (MOPP): Postpartum Tight vs Standard Blood Pressure Control Impact of postpartum tight blood pressure control using remote patient monitoring on emergency department visits for hypertensive disorders and blood pressure throughout the 6 weeks Postpartum. In the graph, the intervention and control groups include blood pressure from all emergency department visits, hospitalizations, or postpartum clinic visits. The intervention group additionally consists of all blood pressure readings from the remote patient monitoring. The shaded areas denote the 95% pointwise confidence bands. ED = emergency department.

In the intervention cohort, participants took median (IQR) of 8 (5-12), 11 (4-14), 7 (2-13), 5 (1-10), 3 (1-8), and 2 (0-6) blood pressure readings during the first, second, third, fourth, fifth, and sixth week postpartum. The first week was truncated since patients were discharged on postpartum day 2.6 ± 1.3. Compliance with blood pressure readings was 64%, primarily driven by a decrease in blood pressure readings with increasing time in the study. On average, medication was adjusted 1.8 times per patient during the study period, and 75% of patients had one or more adjustments to medication during the first week postpartum. Fourteen (3.6%) patients had persistent blood pressure <100/80 mm Hg during the study period, requiring medication discontinuation. There was one syncopal episode in each cohort and no severe adverse events.

## Discussion

This study evaluated the novel intervention targeting a blood pressure goal of <130/80 mm Hg in the postpartum period instead of the current standard of care of <150/110 mm Hg endorsed by American College of Obstetricians and Gynecologists. This tight blood pressure control was associated with a reduction in postpartum emergency department visits by 68%. The intervention group had lower systolic, diastolic, and mean arterial blood pressures at 6 weeks postpartum. There were fewer hospitalizations for hypertensive disorders, and fewer patients received postpartum magnesium sulfate for seizure prophylaxis after initial hospital discharge.

Based on the growing evidence of cardiovascular protection from tighter blood pressure control in the postpartum period, the decision was made to use AHA guidelines for starting or titrating medication.^6^^,^^7^ However, since blood pressure typically spikes on postpartum days 4 to 6 before decreasing,^15^^,^^16^ remote patient monitoring was employed to closely monitor and titrate medication. Although the Management of Postpartum Preeclampsia and Hypertensive Disorders protocol used a lower blood pressure target to reduce or discontinue medication than previous studies, it appeared to be safe and well tolerated by patients. This builds on the evidence of a previously published small pilot trial that showed tight blood pressure control to less than 130/80 mm Hg was safe in the postpartum period.^35^ Nevertheless, whether this is safe without remote patient monitoring has not been determined. Previous studies have utilized patient self-titration,^7^ which may be more difficult in a population with low health literacy. Further studies must determine if a lower blood pressure target is safe without intensive remote blood pressure monitoring.

There is a growing body of evidence that lower blood pressure improves cardiovascular health. A meta-analysis of nearly one million patients showed that reducing systolic blood pressure by 10 mm Hg and diastolic blood pressure by 5 mm Hg reduced coronary heart disease by 22% and 41%, respectively.^11^ In addition to lowering blood pressure, the study intervention also lowered mean arterial blood pressure, which, in some studies, is an even stronger predictor of cardiovascular health.^36^ AHA guidelines currently recommend treating blood pressure to a target of <130/80 mm Hg since numerous studies have shown that this target lowers the risk of coronary heart disease, stroke, and cardiovascular-related death.^12^^,^^37^ Outside of pregnancy, even a modest decrease in blood pressure can substantially reduce cardiovascular disease.^11^ In one of the most extensive studies of postpartum remote patient monitoring, Kitt et al^7^ showed a 6.5 mm Hg systolic and 5.8 mm Hg decrease in diastolic blood pressure at 9 months postpartum with self-titration of antihypertensive medication to a blood pressure goal of <150/100 mm Hg. In addition, there was a decrease in left ventricular mass index in those who had self-titrated medication. A follow-up of this study also showed a reduction in cardiac remodeling, which is thought to be associated with better cardiovascular outcomes.^6^ On average, these patients only took antihypertensive medications for 40 days, so even a short course of antihypertensive medication in the immediate postpartum period has longstanding cardioprotective effects. There could be additional benefits if this trial were repeated with a lower blood pressure target, such as with the Management of Postpartum Preeclampsia and Hypertensive Disorders protocol.

A previous randomized clinical trial examined initiating medication postpartum before hospital discharge for a blood pressure target of <140/90 mm Hg compared to <150/95 mm Hg and showed no difference in composite maternal morbidity outcomes.^38^ However, this study did not examine blood pressure control after discharge from delivery hospitalization. Since blood pressure fluctuates in the immediate postpartum period without frequent titration of medication, the effect of tighter control was likely not detected. This further supports that tighter control should be implemented in the setting of remote patient monitoring studies, such as in Mei et al,^18^ where medication was increased for multiple blood pressures >140/90 mm Hg and showed a reduction in hospital visits for hypertensive disorders. The combination of even tighter blood pressure control and remote patient monitoring was a novel approach in this study.

Other remote patient monitoring programs have shown decreased postpartum blood pressure, improved adherence to postpartum visits, and a narrowing of the disparities gap.^15^ When looking specifically at emergency department visits, there has been conflicting evidence on whether remote patient blood pressure monitoring decreases emergency department visits and improves maternal outcomes.^18^^,^^21^ Given the recent data, it may be that tighter blood pressure control with remote patient monitoring is more likely to improve outcomes.

### Strengths and limitations

This is one of the first studies to evaluate targeting a blood pressure of <130/80 mm Hg during the postpartum period for patients diagnosed with chronic hypertension and hypertensive disorders of pregnancy. The patient population in this study was diverse in ethnic and racial makeup, primarily publicly insured, were, on average, obese, and were in their early thirties, which is reflective of the patients delivering nationwide. This large, multicenter study implemented a novel approach to treating blood pressure postpartum and showed a reduction in the primary outcome of postpartum emergency department visits for hypertension.

Although a propensity score analysis has methodologic rigor, several limitations are inherent to trials conducted in a nonrandomized fashion. In the retrospective cohort, the mean highest blood pressure during pregnancy was higher than in the prospective cohort, and there were fewer patients with superimposed preeclampsia. This may have been partially driven by the changes in prescribing antihypertensive medications during the antenatal period after the Chronic Hypertension and Pregnancy trial.^13^ During the retrospective cohort, the hospital systems transitioned to a new electronic medical record; thus, some data could not be ascertained. Since most of the retrospective cohort had scanned documents, the authors had to depend on billing codes to identify patients. Coders may have been less likely to code for gestational hypertension since there were many fewer patients identified with gestational hypertension in the retrospective cohort. While this study supports tight blood pressure control in the postpartum period, it is also unknown whether it is safe to implement a target of <130/80 mm Hg without remote patient monitoring managed by a physician at all hours of the day. Emergency department visits were chosen as the primary outcome as a surrogate marker for maternal morbidity and mortality. Still, it is unknown if the intervention will decrease the burden of maternal morbidity and mortality.

## Conclusions

In this propensity score analysis of tight blood pressure control compared to usual control in the postpartum period, there was a decrease in postpartum emergency department visits for hypertension. A randomized controlled trial of tight vs standard blood pressure control is needed to determine whether titrating blood pressure medication to a goal of less than 130/80 mm Hg improves outcomes for postpartum patients.

## Funding support and author disclosures

The authors have reported that they have no relationships relevant to the contents of this paper to disclose.
