# Supplementary material for: Management of Postpartum Preeclampsia and Hypertensive Disorders (MOPP): Postpartum Tight vs Standard Blood Pressure Control
Source: JACC Adv. 2025 Jan 31;4(3):101617. doi: 10.1016/j.jacadv.2025.101617 (PMC11891668; doi:10.1016/j.jacadv.2025.101617)
Supplement: Supplemental Tables 1, 2, and 3, and Supplemental Figure 1 [file mmc1.pdf]

Supplemental Table 1
Protocol for adjusting medication for patients in the intervention cohort.

Color	Level	Blood Pressure	Action
Red	Very High	Systolic ≥ 160<br>OR<br>Diastolic ≥ 110<br>OR<br>Symptoms	If symptomatic*, a physician will assess the need for evaluation.<br>Repeat BP in 15 minutes. If confirmed on repeat and asymptomatic, take an additional dose of oral medication (nifedipine ER 30 mg or labetalol 200 mg).<br>At the next scheduled dose, increase medication (increase by nifedipine ER 30 mg daily or labetalol 200 mg BID).[†‡§]
Orange	High	Systolic 130-159<br>OR<br>Diastolic 80-109	Repeat BP in 15 minutes. If confirmed Orange level or higher on repeat OR two Orange/Red range BPs in 48 hours, then increase medication at next scheduled dose (increase by nifedipine ER 30 mg or labetalol 200 mg BID). [†‡§#]
Green	Normal	Systolic 110-129<br>AND<br>Diastolic < 80	No change
Blue	Low	Systolic 100-109<br>AND<br>Diastolic < 80	Repeat BP in 15 minutes. If confirmed on repeat OR second blue/purple range BP in the last 48 hours, then decrease medication at next scheduled dose (decrease labetalol by 200 mg BID or nifedipine ER 30 mg daily).[⁋#]
Purple	Very Low	Systolic < 100<br>AND<br>Diastolic < 80	Repeat BP in 15 minutes. If confirmed on repeat, then discontinue medication.

* Symptoms include persistent headache, visual abnormalities, upper abdominal, retrosternal or epigastric pain, altered mental status, new dyspnea, and seizures.

† For patients on nifedipine ER 30 mg daily, the next increase should be to nifedipine ER 30 mg BID.

‡ For patients that meet criteria for medication increase within 4 hours after of last dose, given an extra dose at that time, then increase at next scheduled dose.

§ Labetalol should be given in twice daily doses. Doses can be divided into TID for symptoms suggesting intolerance including headaches, fatigue, hypotension with high doses or uncontrolled hypertension. If dosing is TID, increase in labetalol should be by 100 mg TID.

⁋ For patients taking labetalol TID, the first step in downward titration should be to switch to BID dosing with a decrease in total daily dose of labetalol.

Amlodipine can be titrated by 2.5 mg daily. Lisinopril can be titrated by doses of 10 mg daily. Enalapril can be titrated by doses of 5 mg daily. Losartan can be titrated by doses of 50 mg daily. Chlorthalidone can be titrated by doses of 12.5 mg daily. Hydrochlorothiazide can be titrated by 12.5 mg daily.

Supplemental Table 2
Associations between tight versus standard blood pressure control (reference) and adverse maternal outcomes based on the inverse probability of treatment weighting method

	Odds ratio (95% confidence interval)	Doubly-robust odds ratio (95% confidence interval)
Emergency room visit for hypertensive disorders (with or without admission)	0.37 (0.25, 0.54)	0.39 (0.26, 0.57)
Antihypertensive medication at discharge	4.88 (4.09, 5.83)	7.39 (5.88, 9.29)
Labetalol	0.54 (0.45, 0.66)	0.55 (0.44, 0.68)
Nifedipine ER	7.89 (6.55, 9.50)	12.0 (9.52, 15.1)
Other	1.66 (1.07, 2.58)	1.95 (1.22, 3.10)
Postpartum visit attendance		
Attended postpartum visits	1.73 (1.23, 2.44)	1.16 (0.78, 1.71)
Currently prescribed antihypertensive medication at Postpartum visit	0.98 (0.77, 1.24)	1.33 (1.03, 1.72)
Hospital readmission for hypertensive disorders	0.10 (0.04, 0.24)	0.10 (0.04, 0.23)
Emergency Department visit for any reason (with or without hospitalization)	0.76 (0.60, 0.96)	0.80 (0.63, 1.02)
Hospital readmission for any reason	0.33 (0.21, 0.53)	0.31 (0.19, 0.50)

Supplemental Table 3
Differences in blood pressure between the tight blood pressure control and standard blood pressure control groups based on the propensity score-matched analysis

Characteristics	Tight blood pressure control (n = 276)	Standard blood pressure control (n = 429)	Mean difference (95% confidence interval)	Adjusted mean difference (95% confidence interval)
Mean blood pressure prior to discharge				
Systolic	128.6 ± 9.7	130.8 ± 10.7	-1.3 (-3.1, 0.5)	-0.5 (-2.1, 1.2)
Diastolic	77.1 ± 8.4	78.4 ± 8.6	-0.4 (-1.9, 1.0)	0.3 (-1.1, 1.7)
Mean arterial pressure	94.6 ± 8.2	95.3 ± 8.6	-0.7 (-2.2, 0.7)	0.0 (-1.3, 1.4)
Mean blood pressure at postpartum visit				
Systolic	123.0 ± 13.2	128.1 ± 14.3	-4.4 (-7.0, -1.9)	-4.4 (6.8, -2.0)
Diastolic	77.3 ± 9.5	80.9 ± 10.2	-3.3 (-5.2, -1.3)	-3.1 (-4.9, -1.2)
Mean arterial pressure	92.6 ± 10.3	96.3 ±10.7	-3.6 (-5.6, -1.7)	-3.5 (-5.4, -1.7)

Supplemental Figure 1

Blood pressure trends in the propensity score matched cohort after discharge to 42 days postpartum. The intervention and control groups include blood pressure from all Emergency Department visits, hospitalizations, or postpartum clinic visits. The intervention group additionally consists of all blood pressure readings from the remote patient monitoring. The shaded areas denote the 95% pointwise confidence bands.

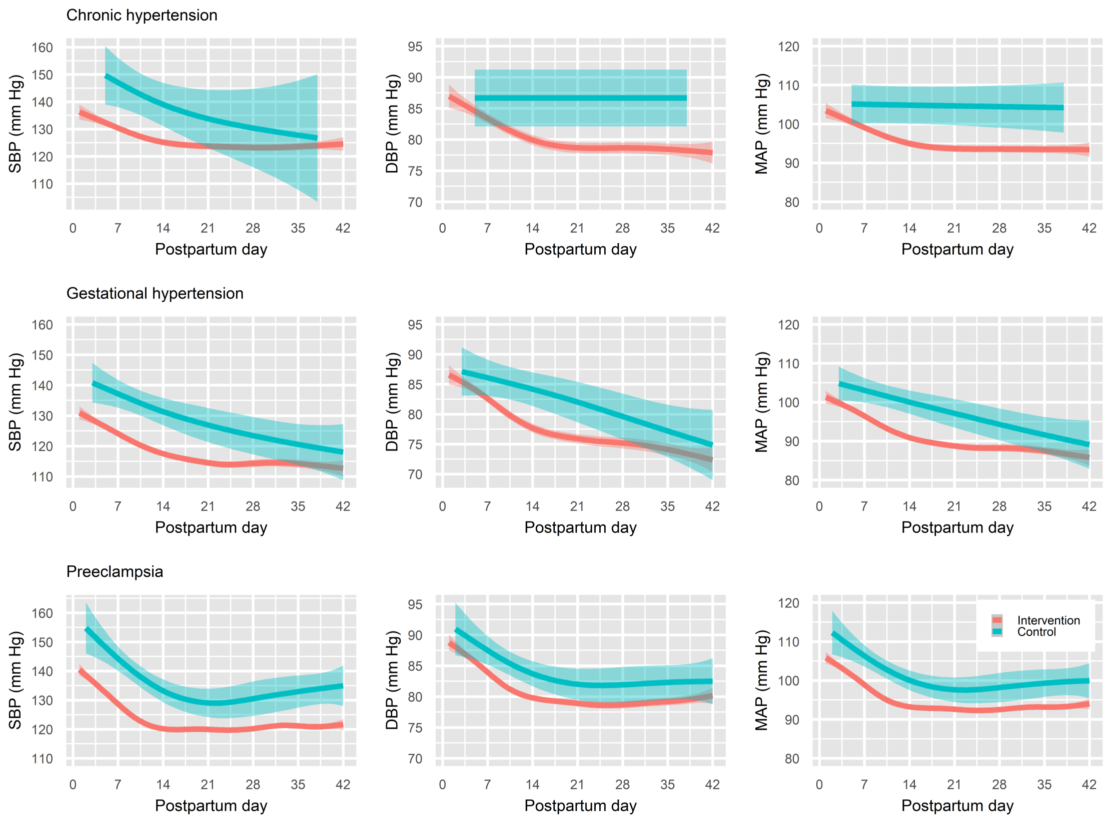
Chronic hypertension
Gestational hypertension
Preeclampsia
SBP (mm Hg)
DBP (mm Hg)
MAP (mm Hg)
Postpartum day
Intervention
Control
